# Mesopancreas excision in pancreatic ductal adenocarcinoma: recurrence patterns — a single-arm descriptive study

**DOI:** 10.1186/s12957-026-04459-4

**Published:** 2026-06-22

**Authors:** Saleh Khairy Saleh, Abdelrhman Gamal Saleh, Tantawi Abdelnaeem Mohamed, Mina Makram Hendy, Mohamed Sadek Farahat, Sara Thabet Boushra, Christina Mosa Kamil, Rabeh Khairy Saleh

**Affiliations:** 1 Department of Surgery, Faculty of Medicine, Minia University, Minya, Egypt; 2 Department of Clinical Oncology, Faculty of Medicine, Minia University, Minya, Egypt; 3 Department of Diagnostic Radiology, Faculty of Medicine, Minia University, Minya, Egypt; 4 Department of Pathology, Faculty of Medicine, Minia University, Minya, Egypt

**Keywords:** Pancreatic ductal adenocarcinoma, Mesopancreas, R0 resection, Lymph node ratio, Recurrence patterns, Survival analysis

## Abstract

**Background:**

Mesopancreas excision has emerged as a critical component of pancreaticoduodenectomy for pancreatic ductal adenocarcinoma (PDAC), potentially improving oncological outcomes. However, comprehensive data on recurrence patterns following mesopancreas excision remain limited.

**Objective:**

To analyze recurrence patterns in patients with PDAC who underwent pancreaticoduodenectomy with mesopancreas excision at a tertiary care center in Egypt.

**Methods:**

This single-arm, single-center, retrospective descriptive cohort study with prospective follow-up included 86 patients with PDAC who underwent pancreaticoduodenectomy with complete mesopancreas excision at Liver and GIT Hospital, Minia University, between February 2018 and February 2024. Patients were followed for a minimum of 24 months, with data collection concluding in February 2026. Data collected included demographics, tumor characteristics, surgical outcomes, pathological findings, and recurrence patterns. The primary outcome was disease recurrence; secondary outcomes included recurrence-free survival (RFS), overall survival (OS), and patterns of recurrence distribution.

**Results:**

Of 86 patients (mean age 58.4 ± 9.2 years, 61.6% male), R0 resection was achieved in 73 patients (84.9%). During follow-up, 52 patients (60.5%) developed recurrence at a median of 11.3 months. Local recurrence occurred in 18 patients (34.6%), distant metastases in 21 patients (40.4%), and combined recurrence in 13 patients (25.0%). The liver was the most common site of distant recurrence, involved in 42.3% of all recurrent cases, followed by lungs (23.1%) and peritoneum (19.2%). Median RFS was 13.2 months (95% CI: 10.8–15.6), and median OS was 18.7 months (95% CI: 16.3–21.1). Lymph node positivity (HR 2.34, *p* = 0.003), poor differentiation (HR 1.89, *p* = 0.012), and elevated CA19-9 (HR 1.67, *p* = 0.028) were independent predictors of recurrence.

**Conclusion:**

Despite complete mesopancreas excision, PDAC demonstrates high recurrence rates with a predominant distant metastatic pattern. Early systemic therapy and intensive surveillance protocols may be warranted for high-risk patients. This study describes the recurrence patterns following complete mesopancreas excision; however, due to the absence of a concurrent control group and the exclusion of patients requiring vascular resection/reconstruction, any superiority in the observed R0 resection rate cannot be directly attributed to the surgical technique itself, and prospective randomized controlled trials remain necessary to establish the oncological benefit of complete mesopancreas excision over standard pancreaticoduodenectomy.

## Introduction

Pancreatic ductal adenocarcinoma (PDAC) is considered one of the most deadly cancers, with a 5-year survival rate less than 10% despite advances in surgical approaches and treatment modalities [1, 2]. The aggressive biological nature of PDAC with systemic spread and invasion at presentation is believed to cause its poor survival outcome [3]. While surgical resection with negative margins (R0), also known as curative resection, is considered an option for tumor cure, recurrence continues to occur at very high rates, between 60% and 85% following complete surgical resection [4, 5].

The mesopancreas concept, similar to that of mesorectum in colorectal cancer surgery, was proposed to improve the efficacy of lymphadenectomy and reduce positive margin rates for pancreatic head cancer [6, 7]. The mesopancreas is defined as retroperitoneal tissue that is bordered anteriorly by the mesenteric vessels and posteriorly by the inferior vena cava and aorta, from the origin of the superior mesenteric artery to the inferior border of the pancreas [8]. It is recognized that this area has lymphatic, nervous, and fat elements that can contain micro-metastatic disease and represents a frequent site of R1 resection in pancreatic head cancer [9].

Several retrospective series and a recent 2024 systematic review and meta-analysis (738 patients across nine studies) have suggested that total mesopancreas excision performed during pancreaticoduodenectomy may increase R0 resection rates, reduce locoregional recurrence, and reduce overall recurrence in selected cohorts [9–11]; however, in the absence of large prospective randomized trials, this conclusion remains controversial, and the magnitude of any survival benefit has not yet been definitively established. The impact of mesopancreas excision on recurrence — either local, distant, or a combination of both — is therefore not well defined, and the recurrence pattern is important for developing appropriate surveillance and adjuvant therapy strategies [12, 13].

With regard to Egypt and the Middle East, there are limited data available regarding the outcome and recurrence rates of pancreatic ductal adenocarcinoma (PDAC) patients [14]. In addition, cultural differences, delay in the onset of symptoms, and differences in the healthcare system may influence the characteristics of the disease in comparison to Western countries [15].

This research aims to undertake a comprehensive analysis of the recurrence patterns in a group of pancreatic ductal adenocarcinoma (PDAC) patients after undergoing pancreaticoduodenectomy with complete mesopancreas excision. We deliberately frame this work as a single-arm, descriptive study of the post-resection recurrence experience: the absence of a concurrent control group of patients undergoing standard pancreaticoduodenectomy without mesopancreas excision is acknowledged at the outset, and the present study is therefore not designed to demonstrate superiority of the mesopancreas excision technique. It is hoped that through an understanding of the recurrence patterns, important information can be gleaned to assist in the postoperative management of a particularly difficult disease.

## Materials and methods

### Study design and setting

This single-arm, single-center, retrospective descriptive cohort study with prospective follow-up was conducted at the Surgery Department, Liver and GIT Hospital, Minia University, Minya, Egypt.

The study included patients who underwent pancreaticoduodenectomy with complete mesopancreas excision for pathologically confirmed PDAC between February 2018 and February 2024. To ensure adequate follow-up, the study analysis was performed in February 2026, allowing a minimum of 24 months follow-up for all patients enrolled in the study. Patients were included if they had at least 24 months of follow-up or documented recurrence before 24 months. As this was a single-arm descriptive study, no concurrent or historical control group of patients undergoing standard pancreaticoduodenectomy without mesopancreas excision was assembled, and consequently the design does not permit direct comparative inference with conventional surgery. The study was approved by the Institutional Ethics Committee (IEC), Minia University Faculty of Medicine Institutional Review Board (MUFMIRB) (approval number: 1118/02/2024), and informed consent was obtained from all patients for treatment and inclusion in the study, in accordance with the Declaration of Helsinki.

### Sample size calculation

Based on previous literature reporting recurrence rates of 70% following pancreaticoduodenectomy for PDAC [4, 5], the required sample size was calculated using the formula for a single proportion: n = [Z² × P × (1 − P)] / d², where Z = 1.96 (95% confidence level), *P* = 0.65 (expected recurrence rate), and d = 0.10 (precision). This yielded a target of 88 patients. During the study period, 86 patients were identified who met all inclusion criteria. This entire cohort was included in the final analysis. Although the final sample size (*n* = 86) was slightly below the calculated target (*n* = 88), this minor discrepancy has a negligible impact on the study’s statistical power and remains adequate for the needs of this single-arm, retrospective descriptive analysis of recurrence patterns.

### Patient selection

#### Inclusion criteria

- Histologically confirmed pancreatic ductal adenocarcinoma
- Tumor location in pancreatic head or uncinate process
- Underwent pancreaticoduodenectomy with complete mesopancreas excision between February 2018 and February 2024
- Age ≥ 18 years
- Complete follow-up data available for a minimum of 24 months (or until death/recurrence)
- ECOG performance status 0–2 at diagnosis

#### Exclusion criteria

- Distant metastases at initial diagnosis (M1 disease)
- Other histological types (neuroendocrine tumors, acinar cell carcinoma, etc.)
- R2 resection (macroscopic residual disease)
- Previous pancreatic surgery or other malignancies
- Death within 90 days postoperatively (perioperative mortality)
- Incomplete pathological or follow-up data
- Requirement for concomitant portal vein (PV) or superior mesenteric vein (SMV) resection and reconstruction.

We acknowledge that the deliberate exclusion of vascular-resection cases removes a high-risk subgroup whose tumours are typically more aggressive and located in close proximity to the superior mesenteric artery; this introduces a selection bias that may have artificially elevated the observed R0 resection rate, and it is for this reason—together with the absence of a concurrent control group—that any superiority in R0 cannot be directly attributed to the surgical technique itself.

### Surgical technique

**Fig. 1:**
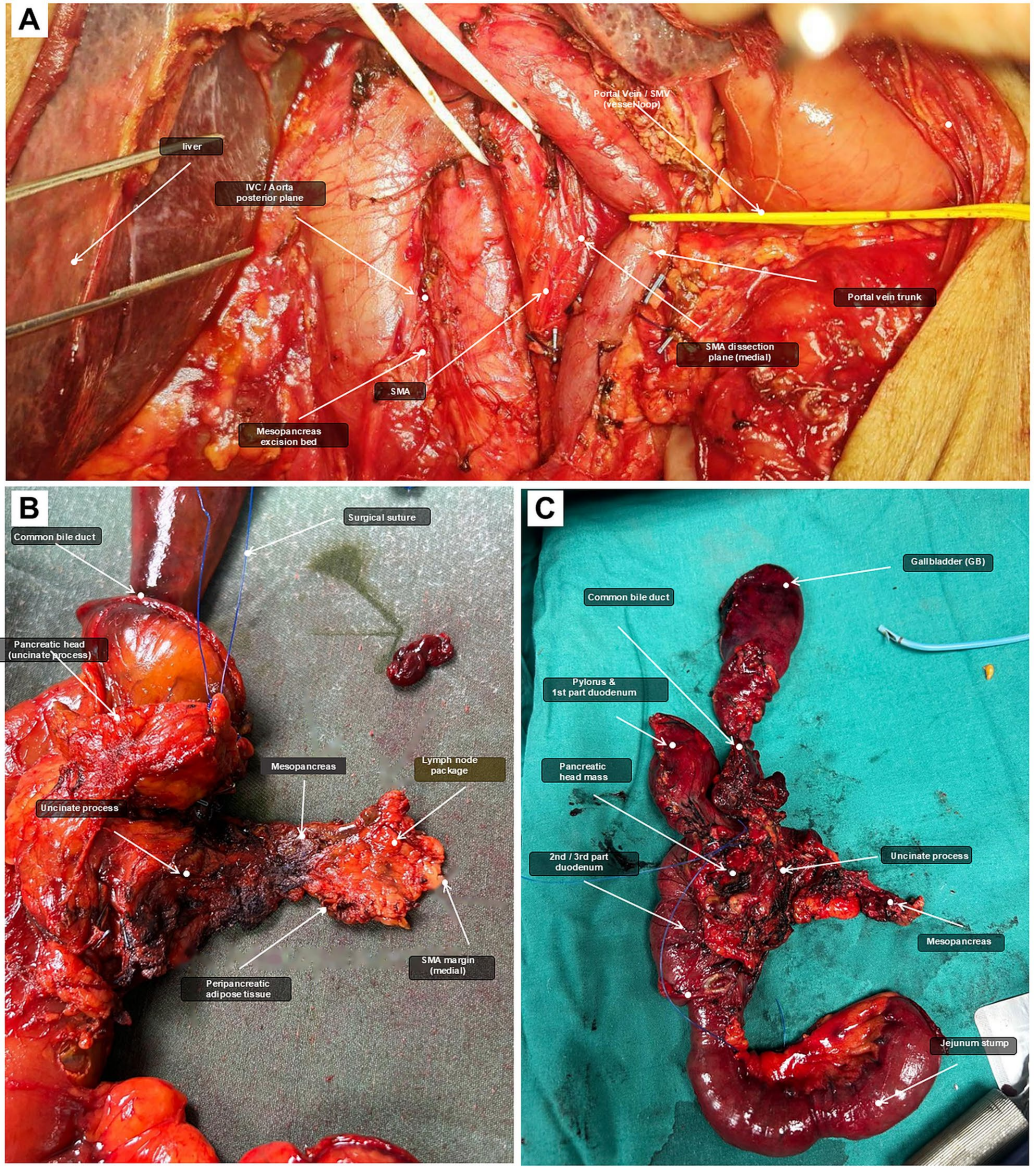
Intraoperative and ex vivo demonstration of Total Mesopancreas Excision (TMPE) during pancreaticoduodenectomy for pancreatic head carcinoma. **A** Operative bed following completion of pancreaticoduodenectomy with total mesopancreas excision (TMPE). The portal vein/superior mesenteric vein (PV/SMV) axis is encircled with a yellow vessel loop; the superior mesenteric artery (SMA) is delineated as the definitive medial boundary of dissection. The mesopancreas excision bed is exposed as the stripped retroperitoneal area posterior to the SMV/PV axis. The posterior boundary — represented by the IVC and aorta adventitial plane — is fully visualized. **B** Back-table photograph of the excised mesopancreas specimen, demonstrating the uncinate process of the pancreatic head in continuity with the mesopancreatic extension — a distinct fibro-fatty, lympho-neural tissue envelope bounded by the PV/SMV anteriorly and the SMA medially. The SMA medial resection margin is identified at the specimen tip, confirming oncologically adequate TMPE. **C** Complete pancreaticoduodenectomy (Whipple) specimen demonstrating all resected components: gallbladder attached via the common bile duct, pylorus and first part of duodenum (pylorus-preserving approach), pancreatic head harbouring the primary carcinoma, second and third parts of the duodenum, jejunum stump, and the mesopancreas demarcated as a distinct component of the en bloc specimen, with the uncinate process in direct continuity with the mesopancreatic tissue. Abbreviations: TMPE = total mesopancreas excision; PV = portal vein; SMV = superior mesenteric vein; SMA = superior mesenteric artery; IVC = inferior vena cava

All procedures were performed by experienced hepatopancreatobiliary surgeons (> 50 pancreaticoduodenectomies annually). The mesopancreas excision technique followed the principles described by Gockel et al. [6] and Peparini et al. [7]. At our institution, the mesopancreas was defined as the fibro-fatty, lymphatic, and neural tissue posterior to the pancreatic head and uncinate process, extending from the right semicircumference of the superior mesenteric artery (SMA) and the superior mesenteric vein/portal vein (SMV/PV) axis anteriorly to the adventitial plane of the inferior vena cava (IVC) and aorta posteriorly, and from the SMA/celiac origin superiorly to the inferior border of the uncinate process and inferior pancreaticoduodenal plane inferiorly. Dissection then proceeded in the avascular plane along the right lateral and posterior aspect of the SMA, with sharp separation of mesopancreatic connective tissue from the arterial adventitia and from the uncinate process. Connective tissue attachments to the retroperitoneum were divided sharply while maintaining an intact specimen and standard regional lymphadenectomy. Cases requiring planned venous resection/reconstruction were not part of this cohort. The mesopancreatic packet was removed en bloc with the pancreatic head specimen. This compartment contains lymphatic channels, adipose tissue, and the right portion of the superior mesenteric neural plexus. Nerve plexus clearance was performed by circumferential skeletonization of the SMA along its right lateral and posterior aspects for 180–270 degrees from its aortic origin, resecting all neural and connective tissue in the mesopancreatic area while carefully preserving the left-sided jejunal branches and inferior mesenteric plexus to minimize postoperative diarrhea. The plane of dissection followed the interface between the peripancreatic fascia and the prevertebral fascia, ensuring en bloc removal of the mesopancreatic package (Fig. 1). Standard pancreaticoduodenectomy was then completed with specimen-oriented pathological assessment of margins. Reconstruction was performed with pancreaticojejunostomy, hepaticojejunostomy, and gastrojejunostomy in standard fashion. The extent of SMA skeletonization was not uniform across the cohort: a right hemicircumferential clearance of approximately 180°, corresponding to the anatomical mesopancreatic compartment as defined by Gockel and Adham, was applied as the default approach, whereas extension up to 270° was performed at the operating surgeon’s discretion when intraoperative assessment raised concern for posterior or medial periarterial tumour extension. A standardized 180° right hemicircumferential dissection was performed in 60 patients (69.8%), whereas extended dissection up to 270° was undertaken in 26 patients (30.2%); this per-case variation is acknowledged as a source of within-cohort heterogeneity and is addressed further in the Discussion.

The completeness of the mesopancreas excision was confirmed postoperatively by macroscopic examination of the gross specimen, including evaluation of whether it constituted an intact, well-circumscribed en-bloc block of tissue with the uncinate process and mesopancreatic compartment in continuity (illustrated in Fig. 1B and C). All specimens were processed using a standardised axial slicing protocol with circumferential margin inking (anterior, posterior, medial SMA-facing, and pancreatic neck/transection margins), and resection-margin status (R0/R1) was assigned according to the Royal College of Pathologists ≥ 1 mm rule. We acknowledge that, unlike the well-validated Quirke grading system used to assess mesorectal completeness in rectal cancer, no equivalent histologically quantifiable grading system currently exists for the mesopancreatic compartment; assessment of completeness therefore relies on combined intraoperative judgement and macroscopic specimen evaluation, which represents an inherent limitation of the technique.

### Data collection

Data were extracted from electronic medical records, operative notes, pathology reports, and outpatient clinic records. Variables collected included:

### Demographics and clinical characteristics

- Age, gender, body mass index (BMI)
- Comorbidities (diabetes mellitus, hypertension, cardiovascular disease)
- Presenting symptoms and duration
- Preoperative CA19-9 levels
- Preoperative imaging findings (CT/MRI)

### Surgical and pathological variables

- Operative time, blood loss, transfusion requirements
- Tumor size and location
- Histological grade (well, moderate, poor differentiation)
- Lymph node status (number examined and positive)
- Lymph node ratio (LNR = positive nodes/total examined)
- Perineural invasion (PNI)
- Lymphovascular invasion (LVI)
- Resection margin status (R0/R1)
- TNM staging (8th AJCC edition) [16]

### Postoperative and follow-up data

- Postoperative complications (Clavien–Dindo classification) [17]
- Length of hospital stay
- Adjuvant chemotherapy regimen and completion
- Follow-up duration
- Recurrence status, site, and timing
- Survival status

### Follow-up protocol

Patients were followed according to a standardized protocol:

- Postoperative clinic visits at 1, 3, 6, 9, 12, 18, and 24 months
- Physical examination and CA19-9 measurement at each visit
- Contrast-enhanced CT scan of chest, abdomen, and pelvis every 3–6 months
- Additional imaging (MRI, PET-CT) performed when clinically indicated

### Recurrence definitions

#### Local recurrence

Tumor recurrence in the pancreatic bed, surgical anastomoses, or regional lymph nodes (peripancreatic, celiac, superior mesenteric).

#### Distant metastasis

Tumor recurrence in distant organs (liver, lungs, peritoneum, bone, brain) or non-regional lymph nodes.

#### Combined recurrence

Simultaneous local and distant recurrence detected at the same follow-up assessment.

Recurrence was confirmed by imaging criteria (RECIST 1.1) [18] or histological confirmation when feasible.

### Statistical analysis

Statistical analysis was carried out using SPSS 26.0 (IBM Corporation, Armonk, NY) and R 4.2.1 software. Continuous variables were expressed as mean and standard deviation or median and interquartile range according to the distribution of the data and were compared by using the two-tailed Student’s t-test or the Mann–Whitney U-test.

Categorical variables were expressed as frequency and percentage and were compared by using the Chi-square test or Fisher’s exact test when applicable. Recurrence-free survival (RFS) was calculated as the time from the date of surgery to the time of first documented recurrence or last follow-up. Overall survival (OS) was calculated as the time from the date of surgery to the time of death or last follow-up. p-values are reported to three decimal places throughout the manuscript, except where the value is extremely small, in which case the convention *p* < 0.001 is used.

**Fig. 2:**
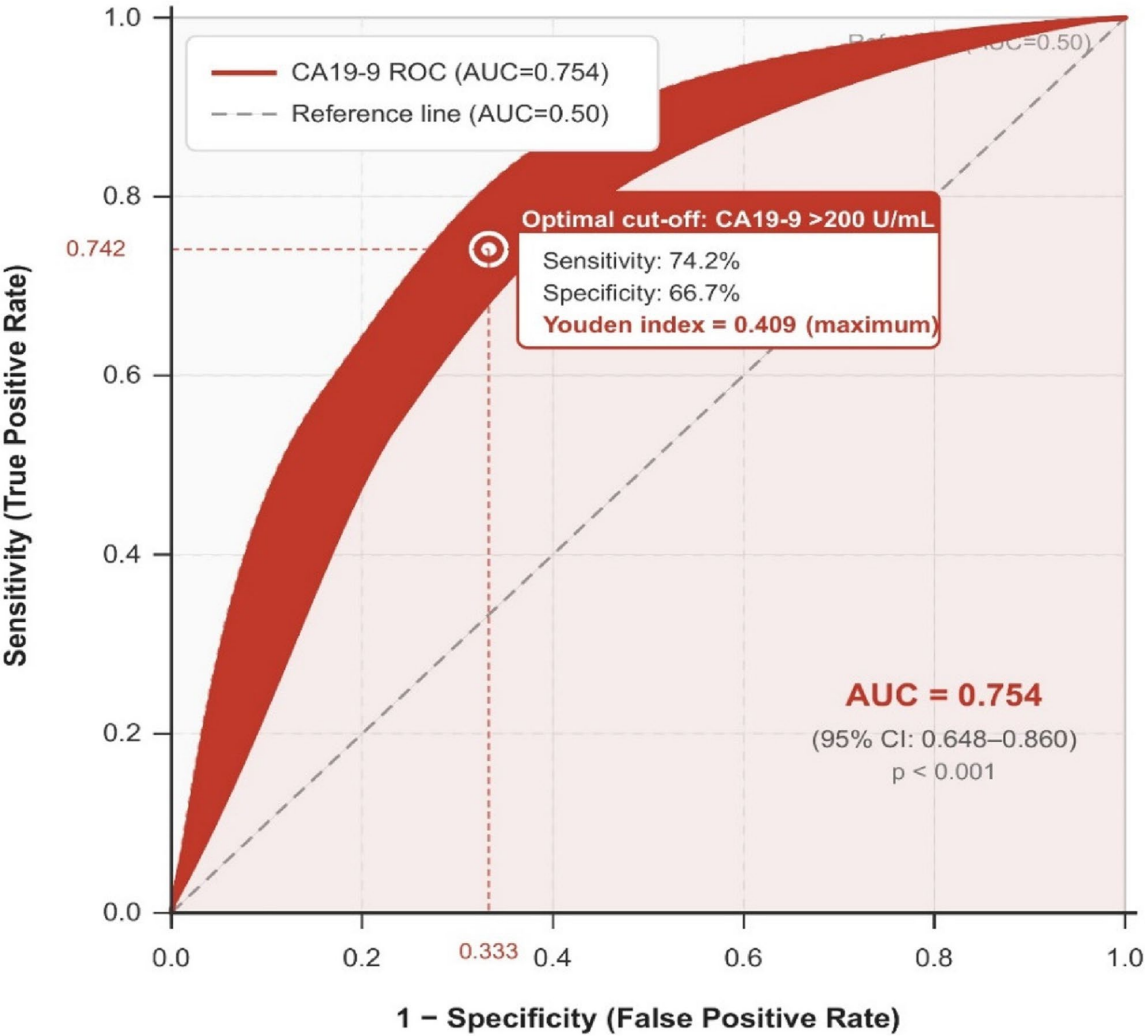
Receiver operating characteristic (ROC) curve for preoperative CA19-9 as a predictor of disease recurrence in 86 patients who underwent pancreaticoduodenectomy with complete mesopancreas excision. The optimal cut-off of 200 U/mL (filled circle) was identified by the maximum Youden index (J = sensitivity + specificity − 1 = 0.742 + 0.667 − 1 = 0.409), yielding sensitivity 74.2% and specificity 66.7%. The area under the curve (AUC = 0.754; 95% CI: 0.648–0.860; *p* < 0.001) indicates good discriminatory performance. The shaded band represents the 95% confidence interval of the ROC curve; the dashed diagonal represents the no-discrimination reference line (AUC = 0.50). Abbreviations: ROC = receiver operating characteristic; AUC = area under the curve; CI = confidence interval; CA19-9 = carbohydrate antigen 19 − 9

Univariable and multivariable Cox proportional hazards regression analyses were performed to identify independent predictors of recurrence. Variables with *p* < 0.10 in univariable analysis were included in the multivariable model. Hazard ratios (HR) with 95% confidence intervals (CI) were reported. Statistical significance was set at *p* < 0.050 (two-tailed). The CA19-9 threshold of 200 U/mL was selected based on ROC curve analysis of our cohort, which identified this value as the optimal cutpoint for predicting recurrence (maximum Youden index; sensitivity 74.2%, specificity 66.7%; Fig. 2), consistent with thresholds validated in comparable published series [19, 20]. This cutoff (200 U/mL) is also supported by Mattiucci et al. [21]. To minimize the recognized confounding effect of biliary obstruction on serum CA19-9 levels, in patients presenting with obstructive jaundice (*n* = 68; 79.1%), the preoperative CA19-9 value used for analysis was the value obtained after biliary decompression (endoscopic biliary stenting or percutaneous transhepatic biliary drainage) and after a documented serum total bilirubin < 2 mg/dL. In the small number of patients in whom CA19-9 was measured before decompression, the most recent post-decompression measurement available prior to surgery was used. To ensure model stability, the multivariable Cox model was restricted using backward stepwise selection with a *p* < 0.10 threshold for variable retention; the dichotomized tumor size variable was removed due to collinearity with T stage, yielding a final model of five variables (events-per-variable ratio = 10.4), consistent with the recommended minimum of 10 events per variable. Tumor size was dichotomized at 3 cm for univariable screening because this threshold approximated the cohort mean and has also been used in prior recurrence analyses [22]. According to the 8th edition AJCC TNM staging system for pancreatic cancer, in which T2 tumours are defined as greater than 2 cm and up to 4 cm in greatest dimension, 3 cm represents the clinically adopted midpoint of this category and is one of the most commonly employed binary cutpoints in published PDAC Cox regression models [16, 23–26].

## Results

### Patient characteristics

**Fig. 3:**
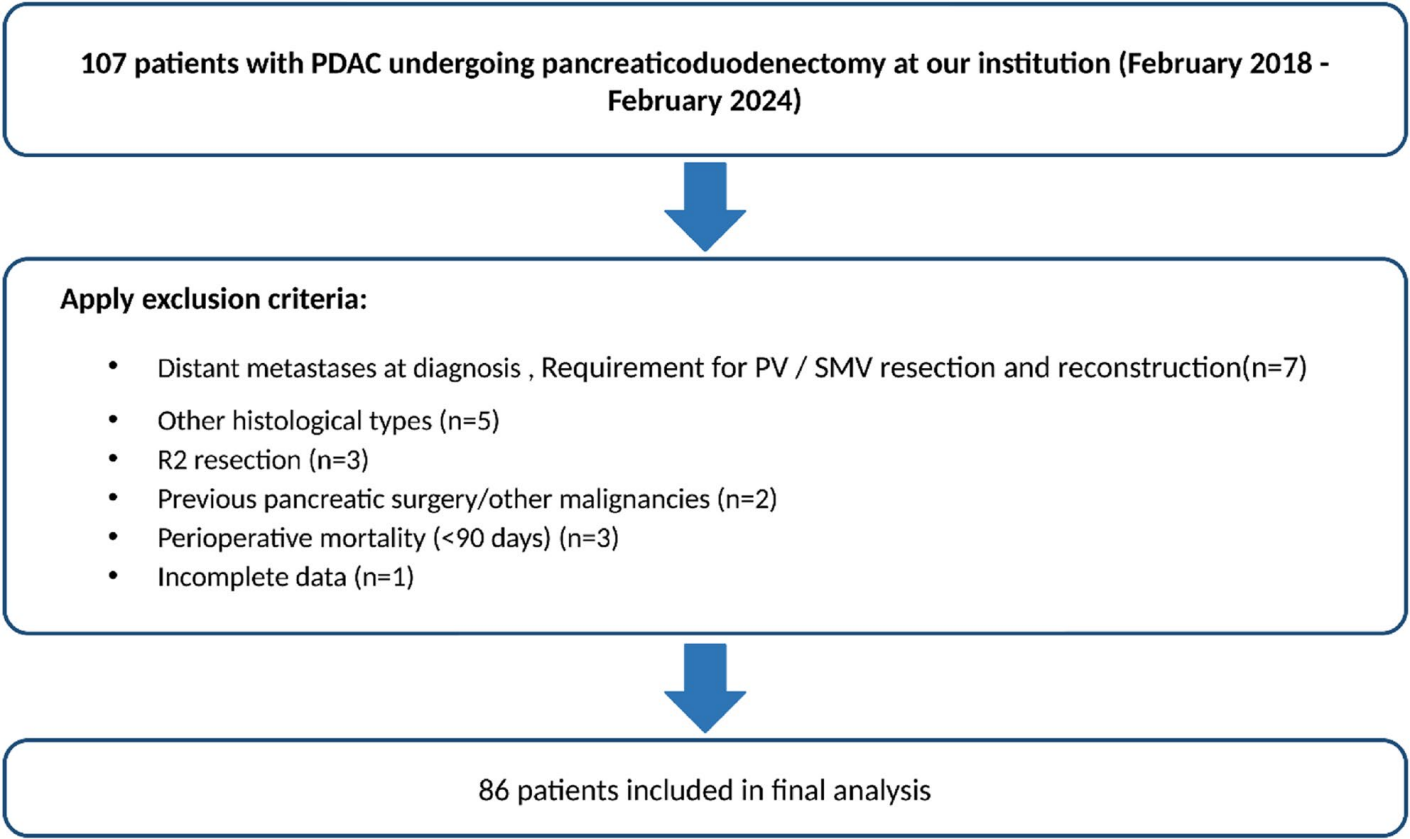
Patient selection flow chart showing screening, exclusion, and inclusion of the final cohort of 86 patients with pancreatic ductal adenocarcinoma who underwent pancreaticoduodenectomy with complete mesopancreas excision between February 2018 and February 2024. Abbreviations: PDAC = pancreatic ductal adenocarcinoma; PD = pancreaticoduodenectomy; TMPE = total mesopancreas excision

Between February 2018 and February 2024, 107 patients underwent pancreaticoduodenectomy for PDAC at our institution. After applying exclusion criteria, 86 patients were included in the final analysis (Fig. 3). The median follow-up duration was 24.0 months (range: 3.2–24.0 months for surviving patients).

Table 1 summarizes the baseline demographic and clinical characteristics. The mean age was 58.4 ± 9.2 years, with male predominance (61.6%). The most common presenting symptom was obstructive jaundice (79.1%), followed by abdominal pain (68.6%) and weight loss (59.3%). Diabetes mellitus was present in 34.9% of patients. Median preoperative CA19-9 level was 287.5 U/mL (IQR: 142.3–523.8).

**Table 1:** Demographics and Clinical Characteristics

Age (years), mean ± SD	58.4 ± 9.2
Age range	38–76
Gender	
Male, n (%)	53 (61.6)
Female, n (%)	33 (38.4)
BMI (kg/m²), mean ± SD	24.8 ± 3.4
Comorbidities	
Diabetes mellitus, n (%)	30 (34.9)
Hypertension, n (%)	41 (47.7)
Cardiovascular disease, n (%)	18 (20.9)
None, n (%)	23 (26.7)
Presenting symptoms	
Obstructive jaundice, n (%)	68 (79.1)
Abdominal pain, n (%)	59 (68.6)
Weight loss, n (%)	51 (59.3)
Nausea/vomiting, n (%)	38 (44.2)
Steatorrhea, n (%)	22 (25.6)
ECOG performance status	
0, n (%)	31 (36.0)
1, n (%)	42 (48.8)
2, n (%)	13 (15.1)
Preoperative CA19-9 (U/mL)	
Median (IQR)	287.5 (142.3–523.8)
< 200, n (%)	32 (37.2)
200–500, n (%)	31 (36.0)
> 500, n (%)	23 (26.7)
Tumor location	
Head, n (%)	58 (67.4)
Uncinate process, n (%)	19 (22.1)
Both, n (%)	9 (10.5)

Abbreviations: *SD* standard deviation, *BMI* body mass index, *ECOG* Eastern Cooperative Oncology Group, *CA19-9* carbohydrate antigen 19 − 9, *IQR* interquartile range, *n* number of patients

### Surgical and pathological outcomes

Surgical and pathological characteristics are presented in Table 2. The median operative time was 385 min (IQR: 340–425), and median blood loss was 650 mL (IQR: 450–900). Thirty-one patients (36.0%) required intraoperative blood transfusion.

**Table 2:** Surgical and pathological characteristics

Operative time (min), median (IQR)	385 (340–425)
Estimated blood loss (mL), median (IQR)	650 (450–900)
Blood transfusion, n (%)	31 (36.0)
Tumor size (cm), mean ± SD	3.4 ± 1.1
Histological differentiation	
Well differentiated, n (%)	11 (12.8)
Moderately differentiated, n (%)	47 (54.7)
Poorly differentiated, n (%)	28 (32.5)
Perineural invasion, n (%)	69 (80.2)
Lymphovascular invasion, n (%)	58 (67.4)
Lymph nodes examined, median (IQR)	18 (15–23)
Positive lymph nodes, median (IQR)	2 (0–4)
Patients with positive nodes, n (%)	61 (70.9)
Lymph node ratio, median (IQR)	0.11 (0–0.22)
Resection margin status	
R0 resection, n (%)	73 (84.9)
R1 resection, n (%)	13 (15.1)
R1 – medial (SMA-facing) margin, n (%)	8 (9.3)
R1 – posterior margin, n (%)	3 (3.5)
R1 – anterior margin, n (%)	1 (1.2)
R1 – pancreatic neck/transection margin, n (%)	1 (1.2)
R1 – bile duct margin, n (%)	0 (0)
AJCC 8th Edition TNM Stage	
T Stage	
T1, n (%)	12 (14.0)
T2, n (%)	28 (32.6)
T3, n (%)	35 (40.7)
T4, n (%)	11 (12.8)
N Stage	
N0, n (%)	25 (29.1)
N1, n (%)	48 (55.8)
N2, n (%)	13 (15.1)
Overall Stage	
IA, n (%)	5 (5.8)
IB, n (%)	8 (9.3)
IIA, n (%)	12 (14.0)
IIB, n (%)	45 (52.3)
III, n (%)	16 (18.6)

Abbreviations: *IQR* interquartile range, *SD* standard deviation, *AJCC* American Joint Committee on Cancer, *TNM* tumour, node, metastasis, *R0* microscopically negative margin, *R1* microscopically positive margin

**Fig. 4:**
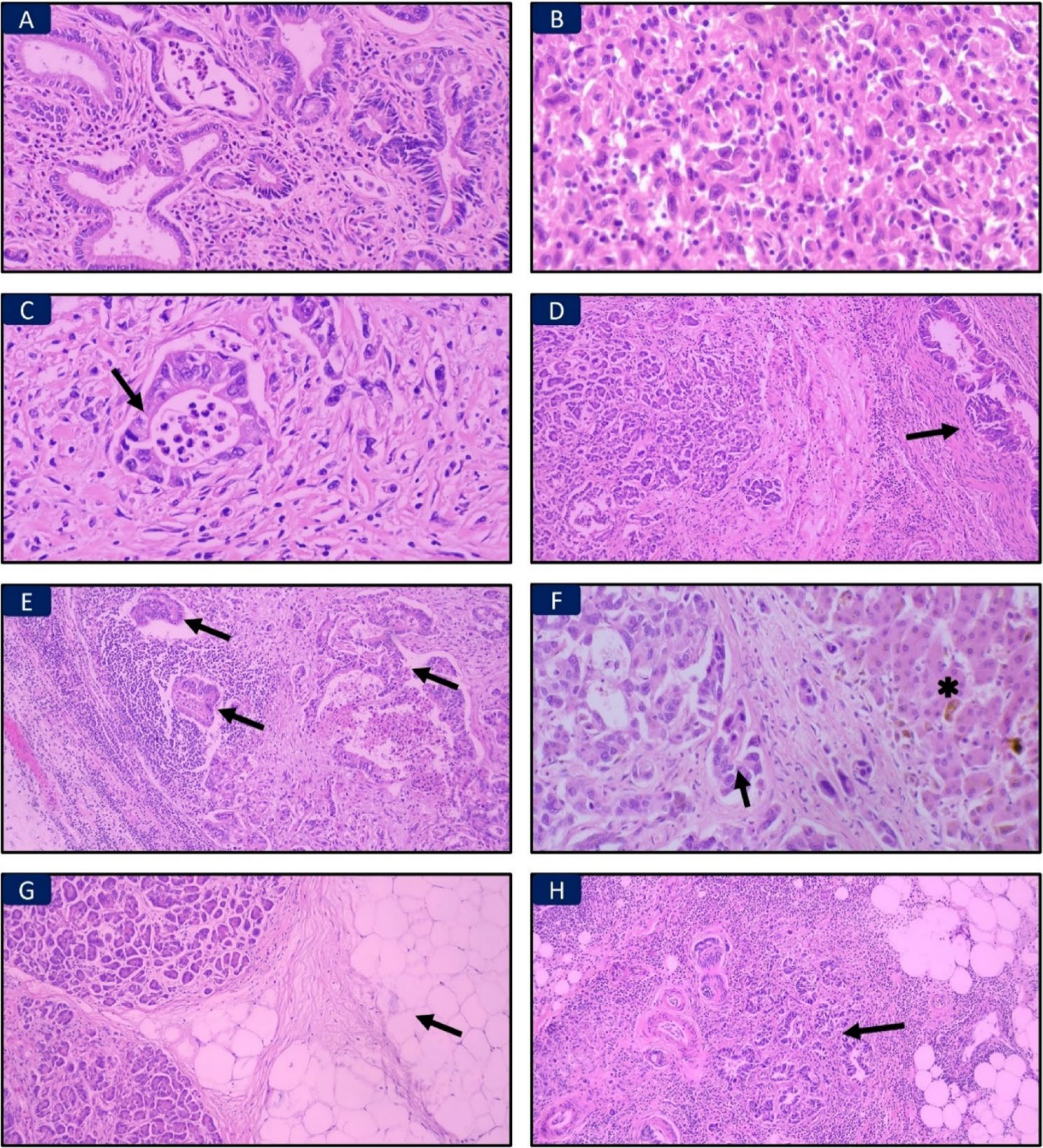
Histopathological findings of pancreatic ductal adenocarcinoma: **A** Well differentiated adenocarcinoma, the tumor showed irregular glands lined by malignant cells (H&E, ×200). **B** Poorly differentiated carcinoma, the tumor showed sheets of malignant cells (H&E, ×400). **C** Vascular invasion by a malignant gland (arrow) was noted (H&E, ×400). **D** Perineural invasion by malignant gland (arrow) (H&E, ×200). **E** Positive lymph node metastasis by malignant glands (arrows) surrounded by remnant of lymphoid tissue (H&E, ×200). **F** Malignant glands (arrow) reach the liver tissue (asterisk) (H&E, ×400). **G** The surrounding fat (arrow) showed no infiltration in the mesopancreas (H&E, ×200). **H** The surrounding fat (arrow) showed malignant infiltration (arrow) in the mesopancreas (H&E, ×200). Abbreviations: H&E = haematoxylin and eosin; × = magnification

Mean tumor size was 3.4 ± 1.1 cm. Histological examination revealed well-differentiated tumors in 11 patients (12.8%), moderately differentiated in 47 (54.7%), and poorly differentiated in 28 (32.5%) (Fig. 4). Perineural invasion was present in 69 patients (80.2%), and lymphovascular invasion in 58 (67.4%).

The median number of lymph nodes examined was 18 (IQR: 15–23), and 61 patients (70.9%) had positive lymph nodes. The median number of positive nodes was 2 (IQR: 0–4), and the median lymph node ratio was 0.11 (IQR: 0–0.22).

R0 resection was achieved in 73 patients (84.9%), while 13 (15.1%) had microscopic positive margins (R1). According to the 8th AJCC TNM staging system [16], 5 patients (5.9%) were stage IA, 8 (9.3%) stage IB, 12 (14.0%) stage IIA, 45 (52.3%) stage IIB, and 16 (18.6%) stage III.

Margin-specific analysis of the 13 R1 specimens identified the medial (SMA-facing) margin as the single most frequent site of positivity, involved in 8 cases (61.5% of all R1 resections; 9.3% of the entire cohort); in all 8 of these specimens the medial margin was the sole positive margin. The remaining R1 designations were attributable to the posterior margin in 3 cases (23.1% of R1 resections; 3.5% of the cohort), the anterior margin in 1 case (7.7% of R1 resections; 1.2% of the cohort), and the pancreatic neck/transection margin in 1 case (7.7% of R1 resections; 1.2% of the cohort); no positivity was recorded at the bile duct margin. Thus the medial (SMA-facing) margin — the margin most directly targeted by mesopancreas excision — accounted for the majority of R1 cases in this cohort (Table 2).

### Postoperative complications

Postoperative complications occurred in 38 patients (44.2%). According to the Clavien–Dindo classification [17]: Grade I in 9 patients (10.5%), Grade II in 16 (18.6%), Grade IIIa in 8 (9.3%), Grade IIIb in 3 (3.5%), and Grade IVa in 2 (2.3%). The most common complications were delayed gastric emptying (15.1%), pancreatic fistula (Grade B/C: 11.6%), and surgical site infection (10.5%). The 90-day mortality rate was 3.5% (3 patients), who were excluded from analysis.

Median hospital stay was 14 days (IQR: 11–19). Seventy-two patients (83.7%) received adjuvant chemotherapy, predominantly FOLFIRINOX (51.4%) or gemcitabine-based regimens (32.3%). The remaining 14 patients (16.3%) did not receive adjuvant treatment: six (42.9%) experienced grade IIIa or higher postoperative complications resulting in performance-status decline precluding timely chemotherapy initiation; five (35.7%) declined treatment after informed counselling citing quality-of-life preference; and three (21.4%) had significant comorbidities (severe cardiovascular disease in two; Child–Pugh B hepatic dysfunction in one) deemed to render chemotherapy high-risk by the multidisciplinary team.

### Recurrence patterns

During the 24-month follow-up period, 52 patients (60.5%) developed disease recurrence. The median time to recurrence was 11.3 months (IQR: 7.8–15.6; range: 3.2–21.8 months). Table 3 details the recurrence patterns and distribution.

**Table 3:** Recurrence patterns and distribution

Overall recurrence, *n* (%)	52 (60.5)
Time to recurrence (months), median (IQR)	11.3 (7.8–15.6)
Recurrence type	
Local recurrence only, n (%)	18 (20.9)
Distant metastasis only, n (%)	21 (24.4)
Combined (local + distant), n (%)	13 (15.1)
Sites of distant metastasis (*n* = 34)	
Liver, n (%)	22 (42.3)
Lungs, n (%)	12 (23.1)
Peritoneum, n (%)	10 (19.2)
Distant lymph nodes, n (%)	8 (15.4)
Bone, n (%)	3 (5.8)
Multiple sites, n (%)	15 (28.8)
Sites of local recurrence (*n* = 31)	
Pancreatic bed, n (%)	23 (74.2)
Regional lymph nodes, n (%)	18 (58.1)
Vascular structures (SMA/SMV), n (%)	12 (38.7)
Surgical anastomoses, n (%)	6 (19.4)
Timing of recurrence	
Early recurrence (≤ 12 months), n (%)	32 (61.5)
Late recurrence (> 12 months), n (%)	20 (38.5)

Abbreviations: *IQR* interquartile range, *SMA* superior mesenteric artery, *SMV* superior mesenteric vein, *n* number of patients

### Recurrence location

- Local recurrence only: 18 patients (34.6% of recurrences; 20.9% of total cohort).
- Distant metastasis only: 21 patients (40.4% of recurrences; 24.4% of total cohort).
- Combined local and distant: 13 patients (25.0% of recurrences; 15.1% of total cohort).

### Sites of distant metastasis (*n* = 34 patients with distant disease)

- Liver: 22 patients (42.3% of all recurrences)
- Lungs: 12 patients (23.1%)
- Peritoneum: 10 patients (19.2%)
- Distant lymph nodes: 8 patients (15.4%)
- Bone: 3 patients (5.8%)
- Multiple sites: 15 patients (28.8%)

### Sites of local recurrence (*n* = 31 patients with local disease)

- Pancreatic bed: 23 patients (74.2% of local recurrences)
- Regional lymph nodes: 18 patients (58.1%)
- Vascular structures (SMA/SMV): 12 patients (38.7%)
- Surgical anastomoses: 6 patients (19.4%)

Table 4 presents the timing of recurrence by pattern. Early recurrence (≤ 12 months) occurred in 32 patients (61.5% of recurrences), while late recurrence (> 12 months) occurred in 20 patients (38.5%). The distant metastatic pattern showed an earlier median recurrence time (9.8 months) compared to local recurrence (13.5 months) (*p* = 0.032).

**Table 4:** Timing of recurrence by pattern

Recurrence Pattern	Median (months)	IQR (months)	Range (months)	Early (≤ 12 mo), *n* (%)	Late (> 12 mo), *n* (%)
Local recurrence only (*n* = 18)	13.5	9.2–17.3	5.2–21.8	8 (44.4)	10 (55.6)
Distant metastasis only (*n* = 21)	9.8	6.5–13.8	3.2–18.4	15 (71.4)	6 (28.6)
Combined recurrence (*n* = 13)	10.2	7.1–14.6	4.8–19.2	9 (69.2)	4 (30.8)
Overall (*n* = 52)	11.3	7.8–15.6	3.2–21.8	32 (61.5)	20 (38.5)

Abbreviations: *IQR* interquartile range, *mo* months, *n* number of patients

### Survival analysis

At the end of follow-up, 43 patients (50.0%) had died. Median recurrence-free survival (RFS) for the entire cohort was 13.2 months (95% CI: 10.8–15.6), and median overall survival (OS) was 18.7 months (95% CI: 16.3–21.1).

**Fig. 5:**
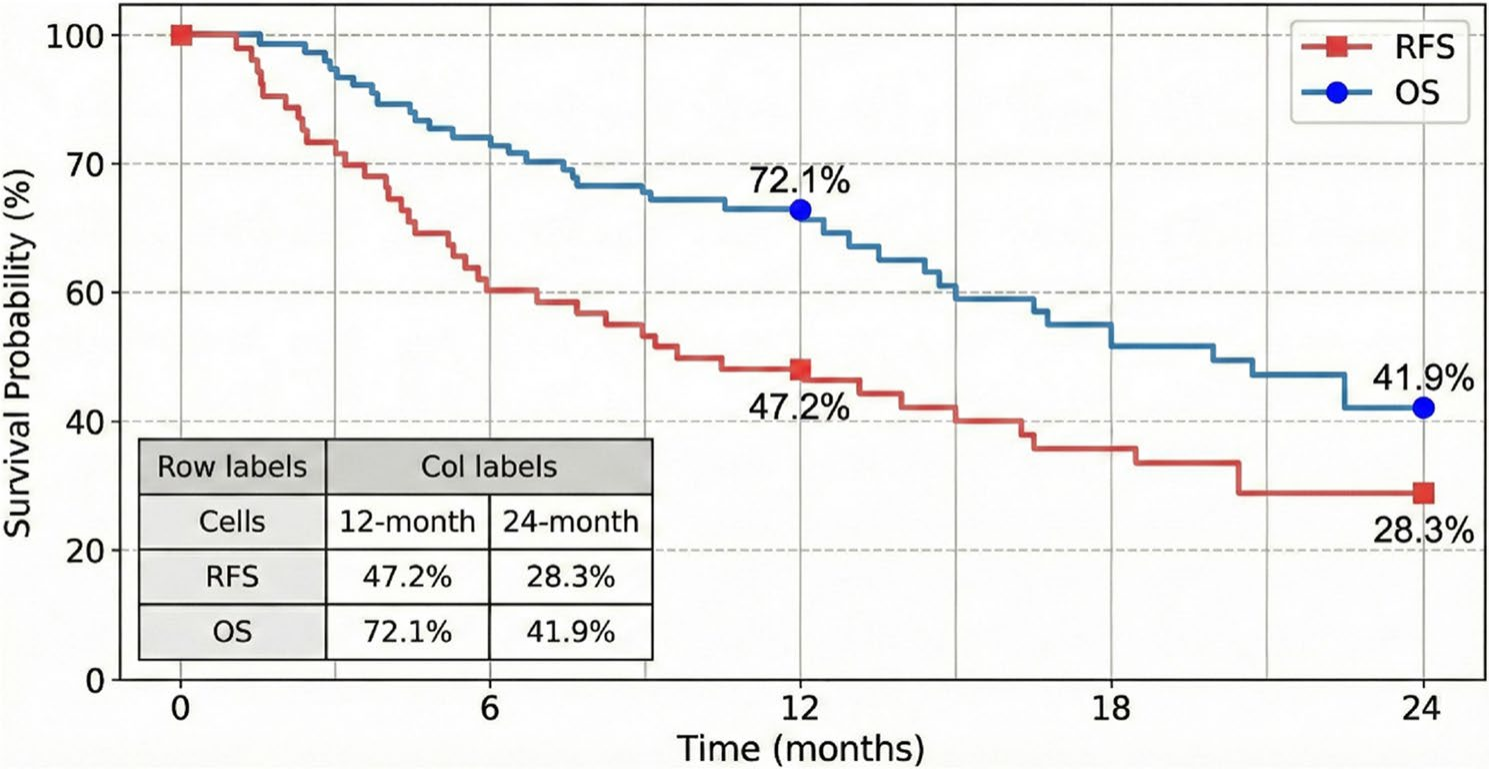
Kaplan–Meier curves for recurrence-free survival (RFS) and overall survival (OS) in the entire cohort (*n* = 86). The 12-month RFS rate was 47.2% and the 24-month RFS rate was 28.3%. The 12-month OS rate was 72.1% and the 24-month OS rate was 41.9%. Abbreviations: RFS = recurrence-free survival; OS = overall survival; KM = Kaplan–Meier; CI = confidence interval; n = number of patients

Figure 5 shows Kaplan–Meier curves for RFS and OS. The 12-month RFS rate was 47.2%, and the 24-month RFS rate was 28.3%. The 12-month OS rate was 72.1%, and the 24-month OS rate was 41.9%.

**Fig. 6:**
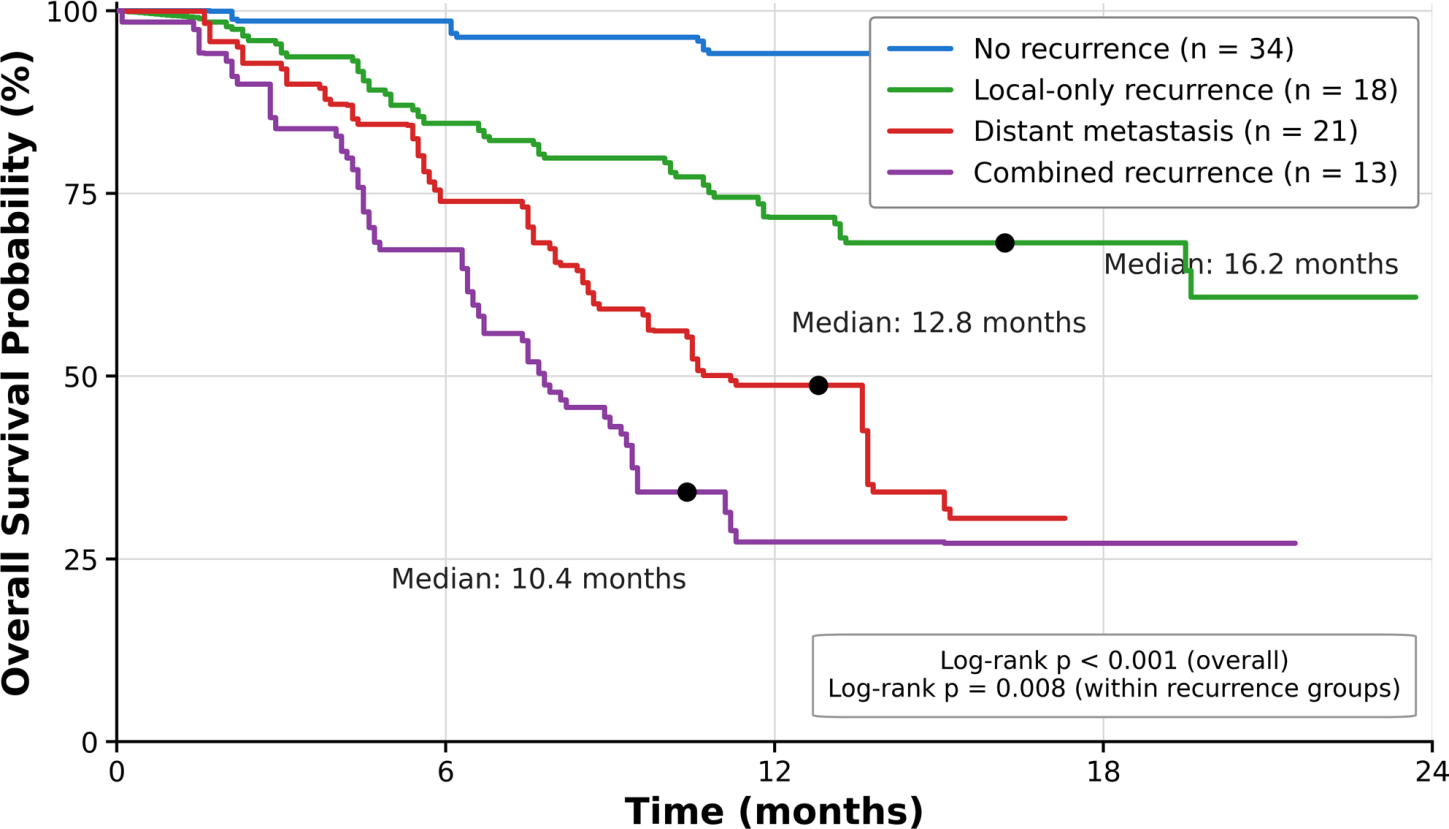
Overall survival stratified by recurrence pattern. Patients with no recurrence had significantly longer survival than those with any recurrence pattern (Log-rank *p* < 0.001). Among patients with recurrence, those with local-only recurrence had better survival (median 16.2 months) compared with distant metastasis (median 12.8 months) or combined recurrence (median 10.4 months) (Log-rank *p* = 0.008). Abbreviations: OS = overall survival; KM = Kaplan–Meier; CI = confidence interval; n = number of patients

Figure 6 displays OS stratified by recurrence pattern. Patients with no recurrence had significantly longer survival than those with any recurrence pattern (*p* < 0.001). Among patients with recurrence, those with local-only recurrence had better survival (median 16.2 months) compared to distant metastasis (median 12.8 months) or combined recurrence (median 10.4 months) (*p* = 0.008). Notably, the median time to recurrence and the median overall survival were nearly identical in the combined recurrence group (10.2 months and 10.4 months, respectively), reflecting a median post-recurrence survival of approximately two weeks. This finding highlights the exceptionally aggressive clinical trajectory of patients who develop simultaneous locoregional and distant relapse: the concurrent systemic and local tumour burden leaves virtually no therapeutic window, conferring a prognosis that is significantly worse than either pattern in isolation (*p* = 0.008).

### Predictors of recurrence

Univariable and multivariable Cox regression analyses for recurrence are presented in Table 5.

**Table 5:** Predictors of Recurrence (Cox Regression)

Variable	Univariable HR (95% CI)	Univariable *p*	Multivariable HR (95% CI)†	Multivariable *p*
Age (> 60 vs. ≤ 60 years)	1.23 (0.71–2.13)	0.461	—	—
Gender (male vs. female)	0.89 (0.52–1.54)	0.666	—	—
BMI (> 25 vs. ≤ 25 kg/m²)	1.34 (0.79–2.27)	0.279	—	—
Diabetes mellitus (yes vs. no)	1.12 (0.65–1.93)	0.679	—	—
Tumor size (> 3 cm vs. ≤ 3 cm)	1.45 (0.84–2.50)	0.182	—	—
Lymph node positivity (yes vs. no)	2.89 (1.52–5.49)	0.001	2.34 (1.35–4.06)	0.003
Lymph node ratio (> 0.20 vs. ≤ 0.20)	2.43 (1.38–4.28)	0.002	—	—
Tumor differentiation (poor vs. well/moderate)	2.12 (1.24–3.63)	0.006	1.89 (1.15–3.10)	0.012
Perineural invasion (yes vs. no)	1.98 (1.08–3.63)	0.027	—	—
Lymphovascular invasion (yes vs. no)	1.87 (1.09–3.21)	0.023	—	—
CA19-9 (> 200 vs. ≤ 200 U/mL)	1.76 (1.03–3.01)	0.038	1.67 (1.06–2.63)	0.028
R1 resection (vs. R0)	2.31 (1.21–4.41)	0.011	1.54 (0.86–2.76)	0.148
T stage (T3/T4 vs. T1/T2)	1.94 (1.12–3.36)	0.018	1.45 (0.84–2.49)	0.182
Adjuvant chemotherapy (yes vs. no)	0.68 (0.35–1.32)	0.257	—	—
Adjuvant regimen type (FOLFIRINOX vs. gemcitabine-based)	0.74 (0.42–1.31)	0.312	—	—

^†^Final multivariable model restricted to 5 variables (EPV ratio = 10.4) using backward stepwise selection: lymph node positivity, poor differentiation, CA19-9 > 200 U/mL, T stage, R1 resection. Perineural and lymphovascular invasion excluded due to EPV constraint. Adjuvant regimen type included in univariable analysis only; subgroup Cox regression was not feasible (~ 22 events in FOLFIRINOX group)

Abbreviations: *HR* hazard ratio, *CI* confidence interval, *BMI* body mass index, *CA19-9* carbohydrate antigen 19 − 9, *R0* microscopically negative margin, *R1* microscopically positive margin, *T* tumour stage, *EPV* events per variable, *FOLFIRINOX* leucovorin, fluorouracil, irinotecan, oxaliplatin, — not entered into multivariable model

#### Univariable analysis

Significant factors associated with increased recurrence risk included the following:

- Lymph node positivity (HR 2.89, 95% CI: 1.52–5.49, *p* = 0.001)
- Lymph node ratio > 0.20 (HR 2.43, 95% CI: 1.38–4.28, *p* = 0.002)
- Poor differentiation (HR 2.12, 95% CI: 1.24–3.63, *p* = 0.006)
- Perineural invasion (HR 1.98, 95% CI: 1.08–3.63, *p* = 0.027)
- Lymphovascular invasion (HR 1.87, 95% CI: 1.09–3.21, *p* = 0.023)
- CA19-9 > 200 U/mL (HR 1.76, 95% CI: 1.03–3.01, *p* = 0.038)
- R1 resection (HR 2.31, 95% CI: 1.21–4.41, *p* = 0.011)
- T3/T4 stage (HR 1.94, 95% CI: 1.12–3.36, *p* = 0.018)

#### Multivariable analysis

Independent predictors of recurrence were:

- Lymph node positivity (HR 2.34, 95% CI: 1.35–4.06, *p* = 0.003)
- Poor differentiation (HR 1.89, 95% CI: 1.15–3.10, *p* = 0.012)
- CA19-9 > 200 U/mL (HR 1.67, 95% CI: 1.06–2.63, *p* = 0.028)

**Fig. 7:**
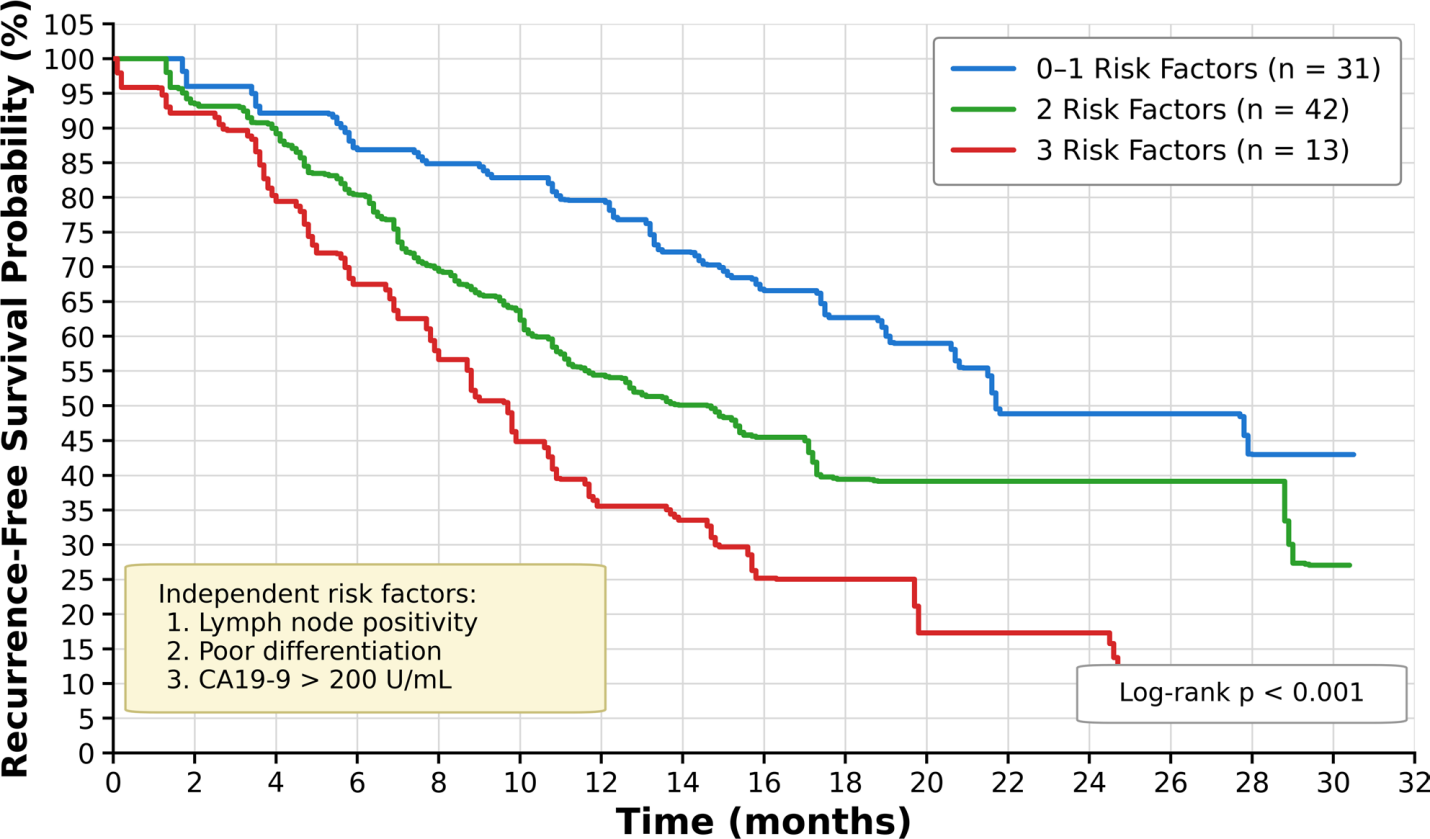
Kaplan–Meier curves for recurrence-free survival stratified by number of independent risk factors (0–1, 2, or 3 factors), demonstrating progressive worsening of RFS with increasing risk-factor burden (Log-rank *p* < 0.001). Risk factors are the three independent predictors identified by multivariable Cox regression: lymph node positivity, poor histological differentiation, and preoperative CA19-9 > 200 U/mL. Abbreviations: RFS = recurrence-free survival; CA19-9 = carbohydrate antigen 19 − 9; HR = hazard ratio; CI = confidence interval; n = number of patients

Figure 7 illustrates survival curves stratified by number of independent risk factors (0–1, 2, or 3 factors), demonstrating progressive worsening of RFS with increasing risk factors (*p* < 0.001).

### Comparison by lymph node status

Table 6 compares characteristics and outcomes between node-negative (N0) and node-positive (N+) groups. Node-positive patients had significantly higher recurrence rates (72.1% vs. 32.0%, *p* < 0.001), shorter median RFS (10.2 vs. 18.9 months, *p* < 0.001), and shorter median OS (15.8 vs. 24.3 months, *p* = 0.002). Distant metastatic pattern was more common in node-positive patients (52.3% vs. 18.5%, *p* = 0.012).

**Table 6:** Comparison by lymph node status

Characteristic	Node-negative (N0) (*n* = 25)	Node-positive (N+) (*n* = 61)	*p*-value
Number of patients, n (%)	25 (29.1)	61 (70.9)	—
Age (years), mean ± SD	57.8 ± 8.9	58.7 ± 9.4	0.682
Male gender, n (%)	14 (56.0)	39 (63.9)	0.498
Tumor size (cm), mean ± SD	2.9 ± 0.9	3.6 ± 1.1	0.009
Poor differentiation, n (%)	5 (20.0)	23 (37.7)	0.113
Perineural invasion, n (%)	17 (68.0)	52 (85.2)	0.084
Lymphovascular invasion, n (%)	13 (52.0)	45 (73.8)	0.061
R0 resection, n (%)	24 (96.0)	49 (80.3)	0.048
CA19-9 > 200 U/mL, n (%)	7 (28.0)	47 (77.0)	< 0.001
Adjuvant chemotherapy, n (%)	20 (80.0)	52 (85.2)	0.548
Recurrence, n (%)	8 (32.0)	44 (72.1)	< 0.001
Local only, n (%)	5 (62.5)	13 (29.5)	0.043
Distant only, n (%)	2 (25.0)	19 (43.2)	0.178
Combined, n (%)	1 (12.5)	12 (27.3)	0.196
Median RFS (months)	18.9	10.2	< 0.001
Median OS (months)	24.3	15.8	0.002
12-month survival, n (%)	22 (88.0)	40 (65.6)	0.024
24-month survival, n (%)	14 (56.0)	22 (36.1)	0.091

Abbreviations: *N0* node-negative, *N +* node-positive, *SD* standard deviation, *CA19-9* carbohydrate antigen 19 − 9, *R0* microscopically negative margin, *RFS* recurrence-free survival, *OS* overall survival, — not applicable

### Site-specific recurrence analysis

**Fig. 8:**
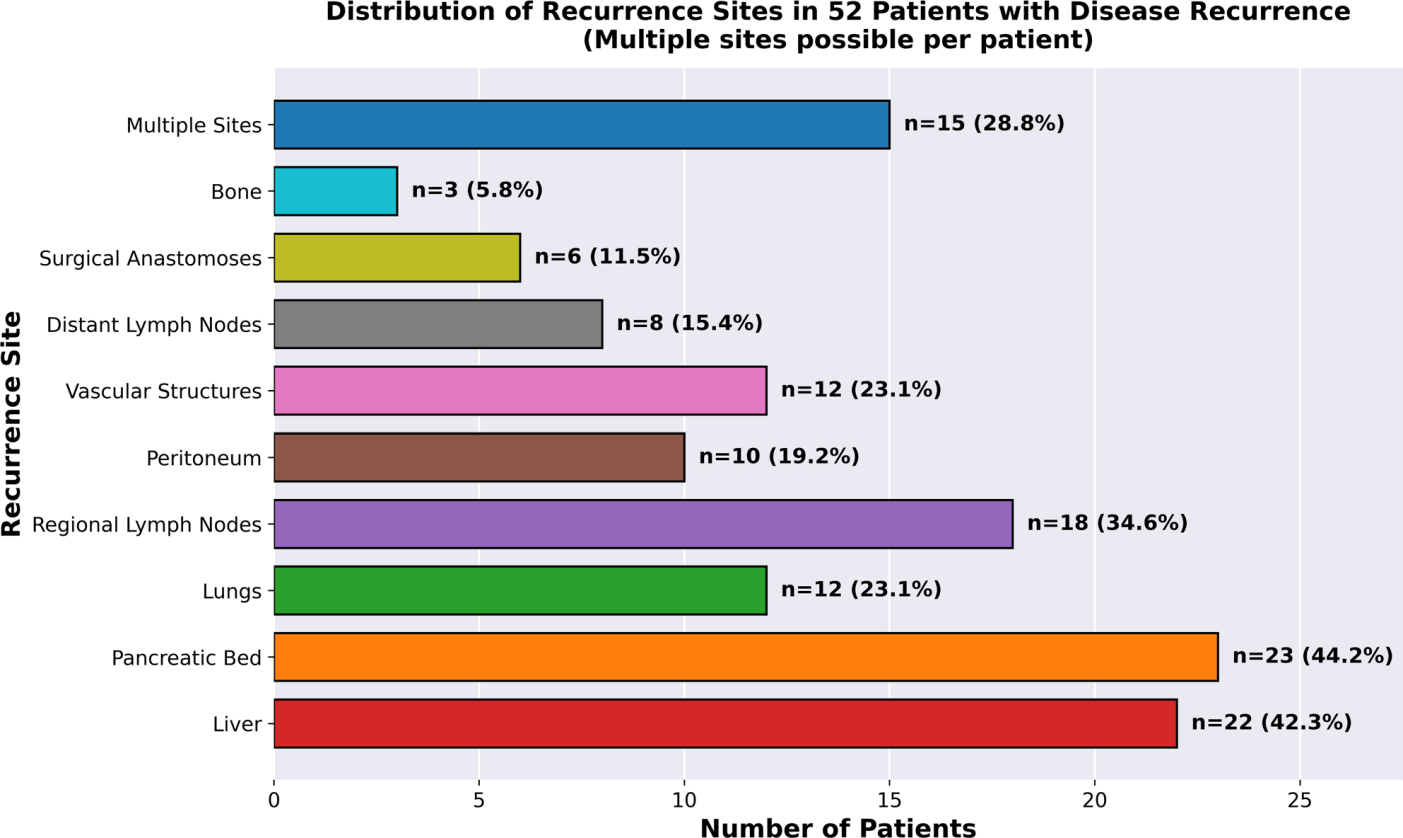
Comprehensive distribution of recurrence sites across all 52 patients with recurrence. The liver was the single most affected organ, involved in 42.3% of all recurrences, highlighting the propensity for haematogenous dissemination despite complete mesopancreas excision. Abbreviations: SMA = superior mesenteric artery; SMV = superior mesenteric vein; LN = lymph node; n = number of patients

Figure 8 presents a comprehensive distribution of recurrence sites across all 52 patients with recurrence. The liver was the single most affected organ, involved in 42.3% of all recurrences, highlighting the propensity for hematogenous dissemination despite complete mesopancreas excision.

**Fig. 9:**
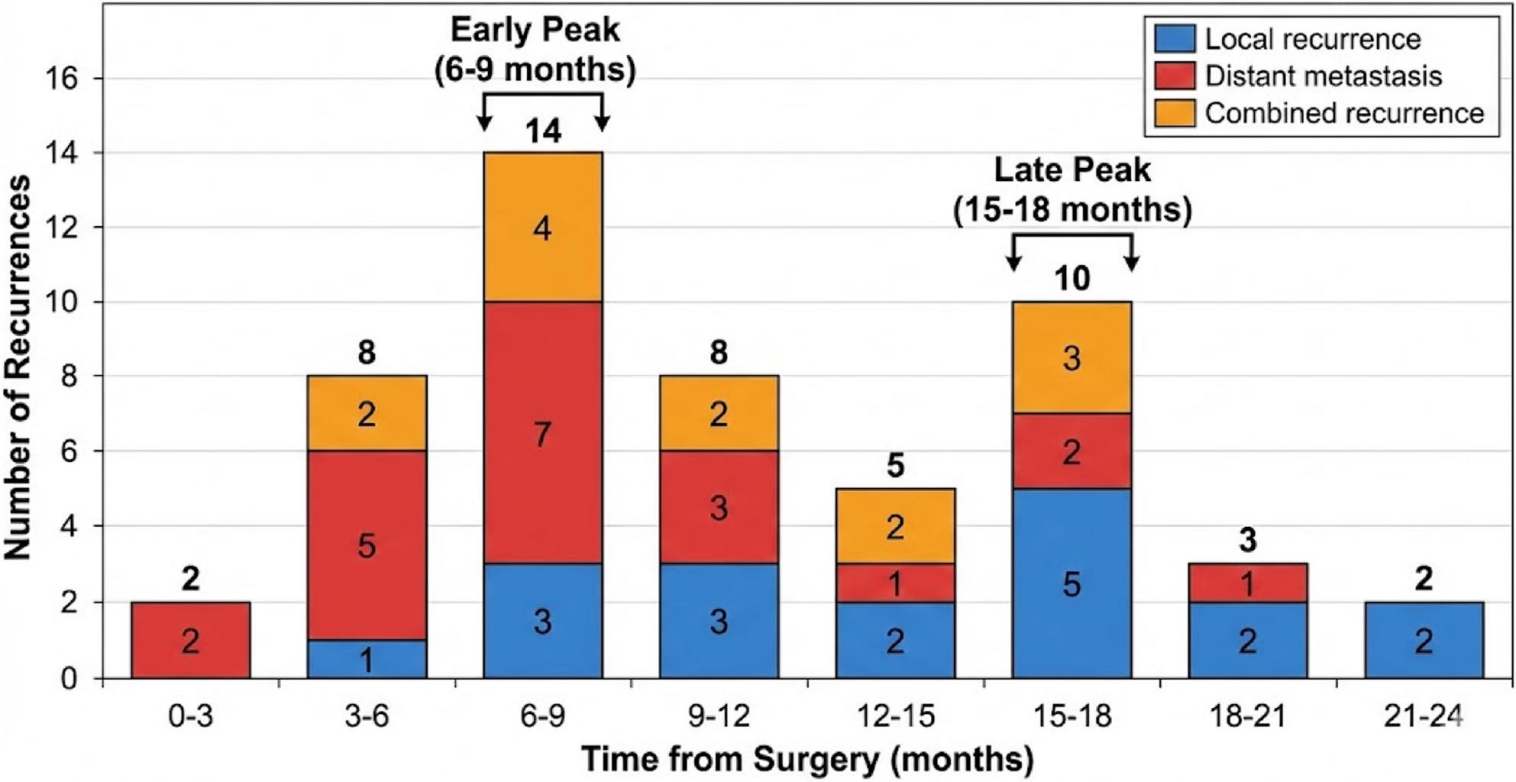
Temporal distribution of recurrences by pattern over the 24-month follow-up period. A bimodal pattern was observed, with peaks at 6–9 months (early recurrence) and 15–18 months (late recurrence). Distant metastases predominated in the early phase, while local recurrences were more evenly distributed throughout the follow-up period. Abbreviations: mo = months; n = number of patients

Figure 9 displays the temporal distribution of recurrences by pattern over the 24-month follow-up period. A bimodal pattern was observed, with peaks at 6–9 months (early recurrence) and 15–18 months (late recurrence). Distant metastases predominated in the early phase, while local recurrences were more evenly distributed throughout the follow-up period.

## Discussion

This retrospective cohort study examined a total of 86 patients with pancreatic ductal adenocarcinoma who underwent pancreaticoduodenectomy with complete mesopancreas excision in a tertiary center. There are several key findings from this study in terms of recurrence patterns and clinical outcome. Although complete resection was achieved in 84.9% of these patients, significantly more than in many previously published series, the recurrence rate was 60.5% at 24 months. This again emphasizes the aggressive nature of this malignancy. The study shows that recurrence in this series of patients occurs more commonly due to distant metastatic sites rather than local recurrence, at a rate of 40.4% and 34.6%, respectively. Liver was found to be the commonest site of metastatic recurrence. Because this was a single-arm, descriptive cohort without a concurrent standard-pancreaticoduodenectomy control group, these findings describe recurrence patterns after mesopancreas excision but do not, by themselves, prove superiority of the technique over conventional surgery.

### Mesopancreas excision and R0 resection rates

The idea of excising the mesopancreas was first proposed by Gockel et al. in 2007 [6], and this can be considered a paradigm shift in the surgical management of pancreatic head cancer. Complete mesopancreas excision involves the systematic removal of the retroperitoneal tissue compartment containing lymphatic and neural tissue, which helps reduce the risk of microscopic residual tumor and improves the outcome for the patient [7, 27]. An R0 resection rate of 84.9% was achieved by our study, which is higher compared to the literature where the range varies from 65% to 85% [23, 28].

Peparini et al. [7] reported an R0 rate of 87% in 78 patients undergoing mesopancreas excision compared to 72% in historical controls without this technique (*p* = 0.030). Similarly, Inoue et al. [10] demonstrated improved R0 rates (91% vs. 79%, *p* = 0.041) and better 3-year survival (38% vs. 25%, *p* = 0.038) with mesopancreas excision in a Japanese cohort of 156 patients. These early studies have been complemented by a 2024 systematic review and meta-analysis (738 patients across nine studies) reporting a pooled R0 resection rate of 87% with total mesopancreas excision and a reduction in locoregional recurrence; however, the authors of that meta-analysis explicitly highlight the heterogeneity of operative definitions, the absence of randomized data, and the persistent uncertainty regarding overall survival benefit [9, 11]. Our results align with these findings, suggesting that meticulous mesopancreas excision can be successfully implemented in an Egyptian setting with outcomes comparable to high-volume centers worldwide.

However, the persistent high recurrence rate despite improved R0 resection highlights a critical paradox in PDAC surgery: achieving negative surgical margins, while necessary, is insufficient to prevent disease recurrence [29, 30]. This suggests that micrometastatic disease is often already present at the time of surgery, even in apparently localized tumors, emphasizing the systemic nature of PDAC [31]. It must be acknowledged that this is a single-arm cohort without a concurrent control group of patients undergoing standard pancreaticoduodenectomy without mesopancreas excision, and that patients requiring vascular resection were excluded a priori. Therefore, although our R0 resection rate of 84.9% compares favourably with published benchmarks, this rate cannot be definitively attributed to the mesopancreas excision technique itself: the exclusion of vascular-resection cases biases the cohort towards anatomically less advanced tumours, and institutional volume effects, perioperative care, and pathological assessment protocols may all contribute. Definitive evidence of any oncological superiority of complete mesopancreas excision over standard pancreaticoduodenectomy will only come from an adequately powered prospective randomized controlled trial.

Taken together with the recurrence pattern observed in our cohort, these data converge on a single message: for tumours with aggressive biological behaviour — in particular node-positive, poorly differentiated disease, and tumours with high preoperative CA19-9 — achieving an R0 local resection alone is insufficient to translate into improved survival. Meticulous local clearance must be combined with effective perioperative and systemic therapy if the natural history of the disease is to be meaningfully altered.

### Recurrence patterns: predominance of distant metastasis

Our finding that distant metastasis (40.4%) exceeded isolated local recurrence (34.6%) contrasts with some earlier studies but aligns with more recent data from high-volume centers performing extensive lymphadenectomy. Groot et al. [12], in a multi-institutional study of 1,629 patients, reported that 75% of recurrences involved distant metastases, with only 12% being isolated local recurrence. Similarly, Jones et al. [13] found that 63% of patients developed distant disease after pancreaticoduodenectomy, compared to 30% with local-only recurrence.

The predominance of distant metastasis in our cohort, despite complete mesopancreas excision, suggests several important implications:

First, the biological aggressiveness of PDAC, characterized by early hematogenous dissemination, may not be significantly altered by improved local surgical technique alone [3, 32]. Rhim et al. [31], using genetically engineered mouse models, demonstrated that pancreatic cancer cells can disseminate to distant organs even before primary tumor formation is complete, providing a biological explanation for the high rate of distant recurrence despite R0 resection.

Second, the high rate of liver metastasis (42.3% of all recurrences) reflects the portal venous drainage of the pancreas and suggests that circulating tumor cells may already have seeded the liver at the time of surgery [33]. Histological studies have also demonstrated micrometastases in the hepatic tissues of patients with PDAC, even in the absence of radiologically evident disease, thereby substantiating this hypothesis.

Third, the 25% rate of combined local and distant recurrence suggests that these two aspects are not mutually exclusive but, rather, often occur simultaneously, which makes it essential to integrate these two aspects in treatment strategies [34]. The predominance of systemic recurrence points to the need to prioritize effective systemic treatment rather than perfecting surgical techniques [35, 36]. Neoadjuvant chemotherapy may also be used to treat micrometastases before surgery, which has shown promising results in recent clinical trials, with many studies showing improved survival rates compared to surgery alone [37, 38].

### Temporal distribution of recurrence

The median time to recurrence was 11.3 months, with 61.5% experiencing recurrence within 12 months post-surgery. This is consistent with current literature, which also reported that the median time to recurrence was within 9–14 months [12, 13, 39]. What is interesting to note is that this curve was bimodal, with peaks at 6–9 months (early recurrence) and 15–18 months (late recurrence) (Fig. 9).

In most cases, early recurrence, as indicated by its occurrence within 12 months, is thought to be associated with aggressive tumor behavior and pre-existing micrometastatic disease at the time of intervention [40]. In our series, distant metastatic patterns were associated with an earlier median recurrence compared with local recurrence (9.8 months vs. 13.5 months, *p* = 0.032).

The patients who experience early recurrence have a much poorer prognosis compared to those who do not experience recurrence, as demonstrated in our survival analysis and other previous studies [12, 22]. Groot et al. proposed that the time to recurrence could be considered an independent prognostic factor; patients who experience early recurrence within 12 months had a median OS of 14.6 months compared with 39.6 months in those who experienced late recurrence after 12 months [22].

The detection of a peak in the late recurrence, as observed in the dataset at 15–18 months, points to the fact that some patients, with relatively less aggressive disease, may enjoy longer periods of disease-free survival. Therefore, these patients can potentially benefit from a more intensified surveillance program after the first year, as current guidelines often advise a reduction in the frequency of surveillance after 12–24 months [41].

### Predictors of recurrence

Our multivariable analysis revealed three predictors for recurrence: lymph node positivity (HR 2.34), poor differentiation (HR 1.89), and CA19-9 > 200 (HR 1.67). These results are consistent with established prognostic factors as they relate to PDAC [19, 20, 23].

#### Lymph node status

Lymph node involvement is one of the most frequently cited poor prognostic indicators for pancreatic ductal adenocarcinoma (PDAC) [19, 23]. Within this patient series, 70.9% were found to have lymph node-positive disease, which is consistent with other studies (65–80%). Patients with lymph node-positive disease were shown to have significantly increased recurrence rates (73.8% vs. 32.0%, *p* < 0.001) and shorter survival (15.8 months vs. 24.3 months, *p* = 0.002) when compared to patients with lymph node-negative disease (Table 6).

The lymph node ratio (LNR) is the proportion of positive lymph nodes to the total number of lymph nodes examined and has been identified as a more precise prognostic factor compared to the simple presence or absence of lymph node positivity. In the univariable analysis, the LNR > 0.20 was associated with a higher risk of recurrence (HR 2.43, *p* = 0.002); however, the effect was not sustained in the multivariable model after adjusting for lymph node positivity [20]. This shows that although the LNR is a prognostic factor, the extent of lymph node positivity is the most powerful prognostic factor.

The prognostic implications of lymph node involvement make it imperative to assess the adequacy of excising the mesopancreas in dealing with nodal disease. Although excision of the mesopancreas addresses the major draining lymph node basins, the presence of positive nodes suggests inadequate clearance or disease extent beyond the mesopancreatic region. The need for an extensive lymphadenectomy beyond the mesopancreas remains debatable, with studies showing no survival benefit but increased morbidity [42].

#### Tumor differentiation

Poor histological differentiation was identified as a predictor for recurrence in the current study, with a hazard ratio of 1.89. Poorly differentiated tumors comprised 32.5% of the total, a figure higher than in some Western studies (20–25%) [43]. This could be due to the delayed presentation and, consequently, the more advanced biological behavior of the tumors in the current population, although the role of genetic and molecular differences cannot be excluded.

Poor differentiation has been found to be associated with increased proliferative potential, increased mutations, and aggressive metastatic potential [32]. The grade of the tumor has also been found to be one of the strongest predictors of survival after resection, with poorly differentiated tumors having a median survival of 12 months, while well-to-moderately differentiated tumors have a survival of 24 months [23].

The association between poor differentiation and distant metastatic recurrence within our data set (58.3% of poorly differentiated tumors and 31.4% of well/moderately differentiated tumors, *p* = 0.015) suggests that tumor biological characteristics are important contributors to systemic recurrence patterns. However, given the absence of a concurrent control group without mesopancreas excision, we cannot determine the relative contribution of surgical approach versus tumour biology to the observed outcomes. These findings nonetheless underscore the need for effective systemic therapies targeting aggressive tumor phenotypes.

#### CA19-9 levels

Preoperative CA19-9 elevation above 200 U/mL was independently related to recurrence (HR 1.67). CA19-9 remains the most commonly used marker in pancreatic ductal adenocarcinoma (PDAC), despite its shortcomings in the form of false positive results in the context of benign biliary obstruction and false negative results in Lewis antigen non-expressers (10% of the population) [19]. Because biliary obstruction is itself an established cause of artefactually elevated CA19-9, all patients in this cohort with preoperative obstructive jaundice and a serum bilirubin > 2 mg/dL underwent biliary decompression (endoscopic or percutaneous) prior to definitive prognostic CA19-9 measurement, and the value used for the prognostic threshold analysis (200 U/mL, identified by the Youden index on ROC analysis) was the post-decompression CA19-9 obtained once the bilirubin had normalised or stabilised. This approach mitigates, although it does not entirely eliminate, the confounding effect of cholestasis on CA19-9 interpretation.

Multiple studies have validated CA19-9 as a prognostic marker in PDAC, with published thresholds ranging from 150 to 500 U/mL depending on the cohort and endpoint studied [19–21]. In our cohort, the threshold of 200 U/mL was chosen because ROC curve analysis identified it as the optimal cutpoint for predicting recurrence (maximum Youden index; sensitivity 74.2%, specificity 66.7%), consistent with contemporary series from comparable populations. Furthermore, normalization of CA19-9 after surgery and its trajectory during adjuvant therapy provide additional prognostic information [44].

In our clinical practice, CA19-9 levels were monitored at each follow-up appointment, and an increase often preceded radiographic evidence of recurrence by 2–4 months. This phenomenon, or biochemical recurrence, can be considered a potential treatment window before clinical evidence of disease reoccurs. However, the best approach to managing rising CA19-9 levels without radiographic evidence of disease is controversial.

### Implications for surveillance and treatment

The recurrence patterns identified in our study have several practical implications for postoperative management:

#### Surveillance strategy

Since the majority of these recurrences, 61.5%, happened in the first 12 months and the pattern of distant metastasis was predominant, the surveillance protocol should focus on systemic imaging rather than the surgical bed alone. The current NCCN guidelines for surveillance are CT scans of the chest and abdomen every 3 to 6 months for 2 years. Our results support these recommendations, although more frequent surveillance, every 3 months, in the first year for high-risk patients, like node-positive, poorly differentiated, and elevated CA19-9, may also be beneficial.

The utility of PET-CT in surveillance also remains controversial. Although PET-CT has the potential to identify recurrence at an earlier stage in some cases, it is not clear whether it is cost-effective and whether it impacts survival. PET-CT was used in this study on a selective basis in situations where conventional imaging was inconclusive, and it was found to identify additional sites of disease in 42.9% of 28 patients in whom it was performed.

#### Adjuvant therapy

This increased rate of recurrence after R0 resection highlights the need for adjuvant chemotherapy. In the current study, the proportion of patients receiving adjuvant chemotherapy was high (83.7%), and the most common regimens were FOLFIRINOX (51.4%) and gemcitabine-based chemotherapy (32.3%). The choice of chemotherapy regimen is based on the performance status of the patients, comorbidities, and patient preference. Of the 72 patients who received adjuvant chemotherapy, 37 (51.4%) received FOLFIRINOX-based regimens and 23 (32.3%) received gemcitabine-based chemotherapy, with the remainder receiving capecitabine monotherapy or investigational regimens per multidisciplinary team decision.

The heterogeneity of adjuvant chemotherapy regimens administered in this cohort — modified FOLFIRINOX, gemcitabine-based regimens, capecitabine monotherapy, and a small group receiving no adjuvant therapy — represents a major potential confounder of the observed recurrence patterns. With only approximately 22 recurrence events in the FOLFIRINOX-treated subgroup, this single-arm cohort is statistically underpowered to determine how different chemotherapy regimens affect recurrence patterns following mesopancreas excision; this important question can only be answered by adequately powered prospective studies in which adjuvant regimen is either standardised or used as an explicit stratification variable. Adjuvant chemotherapy receipt (yes versus no) was included in the multivariable model but did not reach significance (HR 0.68, *p* = 0.257). Future prospective studies with larger sample sizes should stratify analyses by adjuvant regimen type to delineate regimen-specific survival effects.

Recent trials have demonstrated superior survival with FOLFIRINOX or gemcitabine/nab-paclitaxel compared to gemcitabine monotherapy [35, 45]. The PRODIGE 24 trial [35] showed that modified FOLFIRINOX improved median OS to 54.4 months vs. 35.0 months with gemcitabine alone (HR 0.64, *p* = 0.003). However, FOLFIRINOX is associated with significant toxicity, limiting its use in patients with poor performance status or comorbidities.

Given the predominance of distant metastasis in our cohort, intensified systemic therapy may be beneficial. The ongoing debate between neoadjuvant versus adjuvant chemotherapy is relevant here. Neoadjuvant therapy offers theoretical advantages including early treatment of micrometastases, assessment of tumor biology, and potentially higher completion rates [37, 38].

#### Treatment of recurrence

Management of recurrent PDAC is primarily palliative, with median survival after recurrence of 6–12 months. In our cohort, 38 of 52 patients with recurrence (73.1%) received palliative chemotherapy, while 14 (26.9%) received best supportive care due to poor performance status or patient preference.

For isolated local recurrence, retrospective series suggest potential benefit from aggressive local therapies including stereotactic body radiation therapy (SBRT) or even surgical re-resection in highly selected cases. However, in our experience, truly isolated local recurrence was uncommon (34.6% of recurrences), and most patients with apparent local-only disease on initial imaging subsequently developed distant metastases within 3–6 months, suggesting occult systemic disease.

### Comparison with international data

Our results are in general agreement with international norms while also exhibiting distinct characteristics of the patient population in Egypt. The median age of our patient population (58.4 years) is younger than that typically seen in Western populations, which range from 65 to 70 years [1, 2]. This could be due to the younger population demographics in Egypt.

The incidence of jaundice as a presenting feature is significantly high, at 79.1%, which is consistent with other studies and probably reflects the anatomical location of the tumor. However, it is also evident that the median tumor size is 3.4 cm, and 70.9% of patients present with positive nodes, which is probably due to delays in diagnosis, as is characteristic of developing countries [14].

Our R0 resection rate (84.9%) and recurrence rate (60.5%) are consistent with published results from high-volume centers. For example, standardized pathological examination protocols have achieved R0 resection rates of 83% [28], and recurrence rates following pancreaticoduodenectomy range from 60 to 65% in many series [12, 13]. This suggests that our surgical quality and oncological results are consistent with international standards.

The median OS rate of 18.7 months is consistent with the survival rate documented in contemporary literature, which is generally in the range of 16–24 months [23, 35, 45]. Variability in survival may be attributed to differences in patient selection, adjuvant therapy, and follow-up duration. The 2-year survival rate of 41.9% observed in this series is encouraging and likely reflects the combined effects of careful surgical selection, contemporary perioperative care, and adjuvant chemotherapy rather than any single component alone.

### Limitations of mesopancreas excision

Although our results suggest that complete excision of the mesopancreas is technically possible with a high R0 resection rate, the consistently high recurrence rates, particularly for distant metastases, raise questions concerning the long-term impact of this procedure on survival rates. The limitations of this technique should be acknowledged:

First, the technical intricacy of mesopancreas excision increases the duration of surgery (median 385 min in our series). The perioperative morbidity rate could be affected. Although our complication rate (44.2%) is within expected limits for pancreaticoduodenectomy, the incremental benefit over standard pancreaticoduodenectomy is debatable.

Second, there may be anatomical limitations that can prevent the en bloc resection of the mesopancreas. This is more evident in patients with bulky tumors and/or extensive involvement of the mesenteric vessels. In our study, patients requiring vascular resection were excluded to maintain homogeneity of the population. This exclusion introduces a substantial selection bias towards anatomically less advanced tumours, almost certainly contributing to the higher R0 resection rate observed (84.9%) compared with unselected PDAC populations. Clinicians should interpret the R0 rate with this caveat in mind, and future studies inclusive of vascular-resection cases are needed to assess real-world generalizability.

Third, the lack of standardized pathology protocols in the evaluation of the extent of excision of the mesopancreas makes it difficult to verify the extent of excision in different centers. Unlike in the case of total mesorectal excision in rectal cancer, in the evaluation of the extent of excision of the mesopancreas, there are no consensus criteria.

Fourth, the extent of SMA skeletonization in our series was not uniform, ranging from approximately 180° (corresponding to the anatomical mesopancreatic compartment defined by Gockel and Adham) to up to 270° in selected cases. Dissection approaching 270° overlaps conceptually with level 2/3 periarterial nerve plexus dissection (PANP) as characterized by Inoue et al. [10], a procedure that is anatomically distinct from mesopancreas excision in the strict sense. The 2024 systematic review and meta-analysis by da Silva et al. [9] explicitly identifies conflation of these two procedures as a major source of inter-study heterogeneity. This overlap, together with the per-case variation in skeletonization extent within our cohort (approximately 180° in 60 patients and up to 270° in 26 patients), may limit the direct comparability of our findings with published ‘mesopancreas excision’ series in which the dissection was confined to the 180° mesopancreatic compartment.

### Future directions

Several avenues for improving outcomes in resectable PDAC warrant exploration based on our findings:

#### Prospective randomized controlled trial

The core unanswered question raised by our data — what is the actual incremental oncological advantage of complete mesopancreas excision over standard pancreaticoduodenectomy? — can only be resolved by a rigorously designed prospective randomized controlled trial in which patients with resectable PDAC are randomly assigned to (i) standard pancreaticoduodenectomy or (ii) standard pancreaticoduodenectomy plus systematic complete mesopancreas excision, with R0 resection rate and local recurrence-free survival designated as primary endpoints, and overall survival, perioperative morbidity, and pattern of recurrence as key secondary endpoints. Such a trial, ideally multi-centre and stratified by adjuvant chemotherapy regimen, is the necessary next step before any definitive recommendation in favour of routine mesopancreas excision can be made. The ctDNA-based, neoadjuvant, and molecularly stratified strategies discussed below should be considered complementary, not alternative, future research priorities to such a trial.

#### Molecular profiling

This diversity in recurrence patterns points to the biological diversity not fully reflected by conventional clinicopathological characteristics. Molecular types of pancreatic ductal adenocarcinoma (PDAC) like the conventional and basal types have different prognostic and treatment implications [32]. This would help in the development of personalized treatment and surveillance approaches.

#### Circulating tumor DNA (ctDNA)

Emerging evidence suggests that ctDNA can detect minimal residual disease before radiographic recurrence, potentially enabling earlier intervention. Evidence suggests that postoperative ctDNA positivity is a highly sensitive predictor of recurrence, with detection occurring a median of 6.9 months before radiographic recurrence [44]. Implementation of ctDNA monitoring in our setting is awaiting validation and assessment for cost-effectiveness.

#### Neoadjuvant therapy

The high rate of distant recurrence within this group may warrant consideration of chemotherapy regimens aimed at treating micrometastatic disease before surgery. The PREOPANC-1 trial [38] has proved the feasibility of neoadjuvant chemoradiotherapy with gemcitabine-based regimens, although the survival benefit was limited. Trials currently underway, such as those evaluating FOLFIRINOX regimens [46], may help elucidate the best combination of surgery and chemotherapy.

#### Targeted therapies

For individuals with actionable mutations, such as BRCA1/2, PALB2, NTRK, and MSI-high, targeted therapy options, including PARP inhibitors, TRK inhibitors, and immune checkpoint inhibitors, represent new avenues of therapy. Genetic testing, both germline and somatic, should be considered in all individuals with pancreatic ductal adenocarcinoma.

#### Enhanced recovery protocols

Any attempt to reduce postoperative complications may be a contributing factor to the increased rate of completion of adjuvant therapy, which is associated with patient survival. ERAS has been shown to reduce postoperative complications and hospital stay without adversely affecting the oncologic outcomes. The implementation of a robust ERAS protocol is currently under way.

### Study limitations

Several limitations of our study must be acknowledged:

First, with this retrospective method, the possibility of selection bias and information bias occurs. Nevertheless, our data were gathered from an institutionally maintained database with prospective updates and high completeness of follow-up (98.8%).

Second, as a single-center study, it is possible that the general applicability of our findings may be limited, though we are encouraged by the similarities of our case distribution and outcome to published multi-center studies.

Third, it is possible that 24 months of follow-up would miss later recurrences beyond two years. However, most recurrences of PDAC occur within 24 months, and our period of follow-up compares favorably to many studies.

Fourth, there is no concurrent control group that underwent standard pancreaticoduodenectomy without mesopancreas excision. Consequently, we cannot establish a direct causal link between the TME technique and the observed R0 resection rate; institutional volume effects and selection factors may also contribute. A randomized controlled trial would be required to prove superiority of TME over standard surgery. Additionally, with 52 recurrence events, the multivariable Cox model was deliberately restricted to five variables (events-per-variable ratio = 10.4) using backward stepwise selection, consistent with the recommended minimum of 10 events per variable; the three independent predictors identified were stable across model specifications.

Fifth, the variation in adjuvant chemotherapeutic regimens, that is, FOLFIRINOX and gemcitabine-based regimens, might influence recurrence rates and survival. However, the regimens were tailored to individual patients’ needs. Our results demonstrated that the recurrence rates were similar for both FOLFIRINOX-treated and gemcitabine-treated patients in univariable analysis; however, the regimen heterogeneity represents an important potential confounder and limits definitive conclusions regarding the independent effect of adjuvant chemotherapy on recurrence outcomes. Future adequately powered studies should stratify analyses by regimen type.

Sixth, we did not have molecular and genetic data for the cohort, and this limited our ability to relate recurrence patterns to tumor type. Further studies with this type of data will provide greater insights.

## Conclusion

This retrospective single-arm, single-centre, descriptive cohort study of 86 patients with pancreatic ductal adenocarcinoma who underwent pancreaticoduodenectomy with complete mesopancreas excision documented an R0 resection rate of 84.9% and a two-year recurrence rate of 60.5%. Because no concurrent control group undergoing standard pancreaticoduodenectomy without mesopancreas excision was available, and patients requiring vascular resection or reconstruction were excluded a priori, these findings describe the performance of this technique in a single institutional setting and do not permit direct causal attribution of the R0 resection rate to the mesopancreas excision procedure per se. The predominance of distant metastatic recurrence, particularly hepatic involvement in 42.3% of all recurrences, underscores the systemic nature of PDAC even following margin-negative resection.

Lymph node positivity, poor histological differentiation, and preoperative CA19-9 greater than 200 U/mL were identified as independent predictors of recurrence, enabling clinically meaningful risk stratification and individualized postoperative management planning. The predominance of distant over local recurrence in this cohort suggests that tumour biological characteristics are important contributors to systemic relapse. Definitive evidence of any oncological benefit of complete mesopancreas excision over standard pancreaticoduodenectomy can only come from an adequately powered prospective randomized controlled trial, which we identify as the most important next step in this field. These findings nonetheless highlight the critical need for effective systemic therapies targeting micrometastatic disease in patients with resected PDAC.

Our findings support the implementation of risk-stratified postoperative surveillance, with intensive cross-sectional imaging every three months during the first 12 to 18 months for high-risk patients (node-positive, poorly differentiated, or CA19-9 greater than 200 U/mL); prioritisation of early initiation and completion of modern multi-agent adjuvant chemotherapy; and consideration of neoadjuvant systemic therapy in selected patients to address micrometastatic disease prior to surgical resection. Future research incorporating molecular profiling, circulating tumour DNA monitoring, and targeted therapies may enable personalised management strategies to improve outcomes in this challenging disease.

The implementation of complete mesopancreas excision at a tertiary centre in Egypt, with perioperative and oncological outcomes broadly consistent with international benchmarks, suggests that high-quality hepatopancreatobiliary surgery is achievable in resource-limited settings when performed by dedicated, high-volume teams. Continued efforts to optimise multidisciplinary care, expand access to modern chemotherapy regimens, and participate in international research collaborations will be essential to further improve outcomes for Egyptian patients with pancreatic cancer.

## Data Availability

The de-identified dataset underlying this article is available from the corresponding author on reasonable request.
